# Lay Representations of Cancer Prevention and Early Detection: Associations With Prevention Behaviors

**Published:** 2009-12-15

**Authors:** Helen W. Sullivan, Lila J. Finney Rutten, Bradford W. Hesse, Richard P. Moser, Alexander J Rothman, Kevin D. McCaul

**Affiliations:** Dr Sullivan is affiliated with the Cancer Prevention Fellowship Program and the Behavioral Research Program, National Cancer Institute (NCI), National Institutes of Health (NIH).; Behavioral Research Program, Division of Cancer Control and Population Sciences, NCI, NIH; Behavioral Research Program, Division of Cancer Control and Population Sciences, NCI, NIH; Behavioral Research Program, Division of Cancer Control and Population Sciences, NCI, NIH; University of Minnesota, Twin Cities, Minneapolis, Minnesota; North Dakota State University, Fargo, North Dakota

## Abstract

**Introduction:**

The Common Sense Model of illness representations posits that how people think about an illness affects how they try to prevent the illness. The purpose of this study was to determine whether prevention representations vary by cancer type (colon, lung, and skin cancer) and whether representations are associated with relevant behaviors.

**Methods:**

We analyzed data from the Health Information National Trends Survey (HINTS 2005), a nationally representative survey of American adults (N = 5,586) conducted by telephone interview.

**Results:**

Respondents reported that all 3 types of cancer can be prevented through healthy behaviors; however, fewer did so for colon cancer. More respondents reported screening as a prevention strategy for colon cancer than did so for lung or skin cancer. Representations were associated with colon cancer screening, smoking status, and sunscreen use.

**Conclusion:**

Representations of cancer were associated with relevant health behaviors, providing a target for health messages and interventions.

## Introduction

A substantial proportion of cancer deaths could be prevented through changes to health behaviors (1); furthermore, early detection of disease through screening has shown potential to reduce cancer deaths (2). The Common Sense Model of illness representations posits that how people think about an illness affects how they prevent, test for, and treat the illness (3,4). In particular, the Common Sense Model points to the importance of individuals’ representations of the illness’s 1) identity (eg, “What is cancer?”), 2) cause (eg, “Why do people get cancer?”), 3) timeline (eg, “Is cancer an acute or chronic problem?”), 4) consequences (eg, “How painful is cancer?”), and 5) controllability (eg, “Can cancer be prevented?”) (5). Studies have found that controllability representations are predictive of health outcomes (6). Thus, determining what people think about cancer controllability and whether such thoughts relate to health behaviors is critical for developing health communication messages and interventions.

Several studies have used the Common Sense Model to examine chronic illnesses, including cancer (7). Many of the cancer-focused studies examined cancer patients' representations of the causes and consequences of their cancer (8) or how cancer patients perceive the disease differently than do nonpatients (9). A few studies have examined lay representations of cancer controllability and in particular the extent to which cancer can be prevented or detected. For example, 1 study conducted with a community sample in Spain found that 28% of participants thought cancer was related to individual behavior and 36% thought cancer could be avoided (10). In another study, interviews of a small sample of women recruited from medical and public health services revealed pessimistic attitudes about cancer prevention and screening; participants reported that cancer cannot be prevented and that screening detects cancer when it is too late (11). Other studies focused on particular prevention strategies; for instance, studies using the National Health Interview Survey have shown that approximately 70% of people agree that good nutrition can prevent cancer (12,13). These studies conceptualized cancer as a single disease; participants were not asked about their representations of specific cancers.

A meta-analysis confirmed that representations of controllability predict outcomes such as coping, well-being, and health; however, few of these studies focused on cancer (6). Some evidence suggests that representations of cancer prevention may influence cancer-relevant behaviors. For example, in a national survey, believing that people cannot do much to prevent cancer in general was associated with less physical activity and lower fruit and vegetable consumption (14). In one study, believing that skin cancer can be prevented was related to preventive behavior intentions (15), although this relationship is not always found (16). Similarly, data suggest that believing that screening is effective is related to intentions to screen and subsequent screening (17,18).

Because no prior research has systematically studied the Common Sense Model in a nationally representative population, we used data from a national survey to explore people's cancer controllability representations. In addition, we extended prior work on cancer controllability representations by focusing on specific cancers rather than cancer in general. We explored how people think about primary prevention (ie, activities that reduce the risk of disease) and secondary prevention (ie, early detection of disease) for colon, lung, and skin cancer. We first examined whether prevention representations varied by cancer type (colon, lung, and skin cancer). Second, we examined whether prevention representations were associated with cancer-relevant behaviors.

Representations of colon, lung, and skin cancer were included in the survey we used because they are relatively common in the United States, affect men and women, and vary in the extent to which they can be prevented, detected early, and treated (19). There is solid evidence that smoking avoidance and long-term smoking cessation can prevent lung cancer (20). For skin cancer, there is evidence that sun exposure is linked to skin cancer, which supports recommendations that people reduce sun exposure. Although evidence is insufficient to suggest that wearing sunscreen can prevent skin cancer (20), sunscreen use is recommended to the public (21). For colon cancer, insufficient evidence exists for the preventive role of a low-fat, high-fiber diet rich in fruits and vegetables (20); however, increasing fruit and vegetable consumption is recommended to the public for colon cancer prevention (22). Finally, colon cancer screening is strongly recommended for people older than age 50, whereas evidence is insufficient to recommend screening for lung or skin cancer (23).

## Methods

### Data source

Data for our analysis were drawn from the 2005 Health Information National Trends Survey (HINTS 2005) (24). HINTS is a national probability survey of the US adult population conducted by the National Cancer Institute (NCI) every 2 years. The survey is designed to capture the public's cancer-related knowledge, attitudes, and behaviors. Data from HINTS 2005 was collected from February 2005 through August 2005 (http://hints.cancer.gov). HINTS 2005 underwent an expedited review with the NCI's institutional review board in 2004, and clearance was obtained from the US Office of Management and Budget (OMB no. 0925-0538). Respondents (N = 5,586) were selected by using random-digit dialing and completed a 1-time telephone interview administered by trained interviewers. One adult aged 18 or older in each household was selected for the extended interview by a household screener. The final response rate for the household screener was 34%, and the final response rate for extended interview was 61%. Further details about the sample and sampling design are available elsewhere (24).

### Measures

Concurrent protocol analysis techniques were used to evaluate the measures in a cognitive laboratory (25,26). Items were reviewed and modified as necessary to stabilize interpretation and use. The full survey was pilot tested.

### Demographic and health characteristics

Respondents were asked to report their age, sex, race/ethnicity, income, and education. They were asked whether they had ever been diagnosed with cancer and whether any of their family members ever had cancer. We categorized respondents as having no cancer history, family history only, personal history only, or family and personal history.

### Prevention representations

Respondents were randomly assigned to answer prevention representation questions about colon, lung, or skin cancer. If respondents had been diagnosed with the specific cancer they were assigned to, they were not asked any prevention representation questions.

Respondents were first asked an open-ended question about their prevention representations: "What are some things that people can do to reduce their chances of getting colon [lung, skin] cancer?" Responses to this question were coded into several categories that represented the most frequently listed behaviors. For colon cancer, the primary prevention categories were the following: "don't drink alcohol," "don't smoke," "eat fiber," "eat fruits and vegetables," "eat healthy/better nutrition," and "exercise." For lung cancer, the primary prevention categories were the following: "avoid asbestos," "avoid polluted air," "don't smoke/quit smoking," "eat healthy," "exercise," and "stay away from secondhand smoke." For skin cancer, the primary prevention categories were "do not use tanning beds/tanning salons," "stay out of the sun," "wear protective hat/clothing," and "wear sunscreen." For all 3 cancers, the secondary prevention categories were "get screened for cancer/get tested" and "have regular checkups." The responses to this item were summed to create the following indices: 1) total prevention behaviors listed, 2) primary prevention behaviors listed, and 3) secondary prevention behaviors listed.

Next, respondents were asked if they agreed or disagreed with the following statements: "There's not much you can do to lower your chances of getting colon [lung, skin] cancer"; "Colon [lung, skin] cancer is most often caused by a person's behavior or lifestyle"; and "Getting checked regularly for colon [lung, skin] cancer increases the chances of finding cancer when it's easy to treat."

### Health behaviors

To assess colonoscopy/sigmoidoscopy screening, respondents aged 45 or older were asked whether they had ever had a colonoscopy or sigmoidoscopy. Respondents who responded affirmatively were asked when they had their most recent colonoscopy or sigmoidoscopy. These 2 items were combined to categorize respondents as having a colonoscopy/sigmoidoscopy "never," "more than 5 years ago," or "5 or fewer years ago." To be consistent with current guidelines, we restricted analyses with this variable to respondents aged 50 or older (27).

To assess fruit and vegetable consumption, respondents were asked a series of questions to discern how often they ate fruit, fruit juice, vegetables, and potatoes during the past month. Responses to these questions were summed to determine servings per day, which was then categorized as none, fewer than 5 servings per day, and 5 or more servings per day (meeting current guidelines) (22).

To assess smoking status, respondents were asked whether they had smoked at least 100 cigarettes in their entire lives. Respondents who responded affirmatively were asked whether they currently smoke cigarettes. These 2 items were combined to categorize respondents as never, former, and current smokers (28).

To assess sunscreen use, respondents were asked to report on a 5-point scale (1 = always to 5 = never) how often they wear sunscreen when they go outside for more than 1 hour on a warm, sunny day.

### Analyses

To account for the complex sample survey design, statistical analyses were conducted by using SUDAAN version 9 (Research Triangle Institute, Research Triangle Park, North Carolina) and used weighting and jackknife variance estimation. Bivariate logistic regression models were conducted to determine whether respondents who completed the colon, lung, and skin cancer sections of the survey differed from each other on demographic or health characteristics. Logistic and linear regressions were conducted to determine whether prevention representations differed among the 3 cancer types after adjusting for demographic and health characteristics. We examined pair-wise comparisons to test for differences between cancer types, using Bonferroni adjustment for multiple comparisons (*P* < .016). Logistic and linear regressions were conducted to determine whether health behaviors were associated with prevention representations after adjusting for demographic and health characteristics. These analyses were specific to each cancer. For colon cancer, the associations between prevention representations and fruit and vegetable consumption and between prevention representations and colonoscopy/sigmoidoscopy screening were tested. For lung cancer, the association between prevention representations and smoking status was tested. For skin cancer, the association between prevention representations and sunscreen use was tested. For each behavior, we examined pair-wise comparisons to test for differences between different groups (eg, current vs former smokers) and adjusted our significance level with Bonferroni test (*P* < .016 for colonoscopy or sigmoidoscopy screening, fruit and vegetable consumption, and smoking status comparisons; *P* < .005 for sunscreen use comparisons). Satterthwaite-adjusted *F* tests and their corresponding *P* values are presented to indicate statistical significance.

## Results

### Demographic and health characteristics

There were no significant differences on any demographic or health characteristics across the cancer type sections (Table 1). Overall, most respondents were female (51.9%), non-Hispanic white (69.9%), educated beyond high school (55.6%), and had a mean age of 45 years. Most (71.4%) reported a family history of cancer, whereas few (11.4%) reported a personal history of cancer.

### Prevention representations across cancer types

Respondents in the colon cancer group listed the fewest total and primary prevention behaviors, followed by respondents in the lung (*F* = 122.17, *P* < .001) and skin cancer (*F* = 336.77, *P* < .001) groups (Table 2). Conversely, respondents in the colon cancer group listed the greatest number of secondary prevention behaviors, followed by respondents in the lung and skin cancer groups (*F* = 234.65, *P* < .001).

The proportion of respondents in the colon cancer group who disagreed that there is not much you can do to lower your chances of getting cancer was significantly smaller than the proportion in the skin cancer group who disagreed (*F* = 6.05, *P* = .005) (Table 2). Similarly, the proportion of respondents in the colon cancer group who agreed that behavior causes cancer was significantly smaller than the proportion in the lung and skin cancer groups who agreed (*F* = 108.93, *P* < .001). The proportion of respondents who agreed that screening leads to early detection was uniformly high across all cancer types (*F* = 1.34, *P* = .27).

### Associations between prevention representations and health behaviors

For colon cancer, screening by colonoscopy/sigmoidoscopy was related to the number of total colon cancer prevention behaviors listed, the number of primary colon cancer prevention behaviors listed, and the number of secondary colon cancer prevention behaviors listed (*F* = 9.57, *P* < .001; *F* = 6.98, *P < *.003; *F *= 3.53, *P =* .04, respectively) (Table 3). Specifically, respondents who were screened by colonoscopy/sigmoidoscopy in the past 5 years listed more colon cancer prevention behaviors overall and more primary prevention behaviors than did those who had never been screened by colonoscopy or sigmoidoscopy (*F* = 13.38, *P* < .001; *F* = 10.04, *P* = .003). A similar trend for secondary colon cancer prevention behaviors (*F* = 5.14, *P* = .03) was noted. No other colon cancer prevention representation questions were related to screening by colonoscopy/sigmoidoscopy (Tables 3 and 4). Fruit and vegetable consumption was not related to any colon cancer prevention representation questions (Tables 3 and 4).

For lung cancer, smoking status was related to the total number of lung cancer prevention behaviors listed (*F* = 5.58, *P* = .01) (Table 3). Specifically, never and former smokers listed more lung cancer prevention behaviors overall than did current smokers (*F* = 7.86, *P* = .007; *F*= 8.60, *P* = .005, respectively). The same was true for the number of primary lung cancer prevention behaviors listed (*F* = 4.96, *P* = .01) (Table 3). Never and former smokers listed more primary prevention behaviors for lung cancer than did current smokers (*F* = 6.35, *P* = .01; *F* = 7.67, *P* = .008, respectively). Smoking status was related to agreeing that behavior causes cancer (*F* = 4.97, *P* = .01) (Table 4). Specifically, never smokers were more likely to agree that behavior causes lung cancer than were current smokers (*F* = 9.12, *P* = .004). No other lung cancer prevention representation questions were related to smoking status (*P* > .05) (Tables 3 and 4).

For skin cancer, sunscreen use was related to the number of skin cancer total prevention behaviors listed and the number of skin cancer primary prevention behaviors listed (*F* = 4.53, *P* = .004; *F* = 3.50, *P* = .01, respectively) (Table 3). Specifically, respondents who never used sunscreen reported fewer skin cancer prevention behaviors overall than did those who reported using sunscreen sometimes or often (*F* = 10.69, *P* = .002; *F* = 15.76, *P* < .001, respectively). Similarly, respondents who never used sunscreen reported fewer primary prevention behaviors for skin cancer than did those who reported using sunscreen often (*F* = 13.08, *P* < .001). No other skin cancer prevention representation questions were related to sunscreen use (*P* > .05) (Tables 3 and 4).

## Discussion

We examined whether people thought about prevention and early detection differently across 3 types of cancer (colon, lung, and skin cancer). Consistent with the current evidence in prevention and early detection, respondents reported that all 3 types of cancer can be prevented through healthy behaviors, but fewer respondents did so for colon cancer. In line with the current evidence base for screening (ie, there is solid evidence for colon but not lung or skin cancer screening) (23), more respondents spontaneously reported screening as a prevention strategy for colon cancer than for lung or skin cancer. However, when asked directly about early detection, most respondents agreed that screening leads to early detection for all 3 cancers; in fact, nearly 90% of all respondents expressed a belief in the value of screening. Thus, the open-ended responses were more in line with state-of-science evidence, whereas the closed-ended responses reflected the belief that screening is uniformly helpful.

Second, we examined whether cancer prevention representations were associated with cancer-relevant behaviors. Similar to past studies (6), these representations were related to health behaviors. Respondents who had recently been screened for colon cancer listed more primary prevention behaviors for colon cancer than did never screeners. Never smokers were more likely to agree that behavior causes lung cancer than were current smokers, and never and former smokers listed more primary prevention behaviors for lung cancer than did current smokers. Respondents who used sunscreen often reported more primary prevention behaviors for skin cancer than did never sunscreen users. The results for colon cancer, in particular, support the idea that lay representations of controllability are associated with related health behaviors (5): respondents were most likely to think of colon cancer as detected early through screening and least likely to think of colon cancer as prevented through healthy behaviors. In turn, representations of colon cancer were related to screening but not to fruit and vegetable consumption. In addition, the pattern of results indicates that respondents who had fewer healthy behaviors reported fewer primary prevention behaviors. This finding suggests that people who engage in unhealthy behaviors may be unaware of current cancer prevention information or may be aware of this information but do not accept it; these people may need targeted messages or interventions focused on cancer prevention and early detection.

Though the current study tests the relationship between prevention representations and behaviors by using nationally representative data, several limitations must be addressed. For example, in using survey data, we had only self-report measures of health behaviors. Health behaviors were measured before prevention representations, which may have primed people to think about these behaviors when answering the prevention representation questions. In addition, the overall response rate for HINTS 2005, although comparable to that of other national telephone surveys, reflects a decline in response rates (29).

In using cross-sectional data, we cannot conclude that our findings are causal and, therefore, we do not know whether prevention representations influence behavior or whether engaging in healthy behaviors influences prevention representations. Because the relationship between cognition and behavior is often reciprocal, changes in prevention representations may lead to changes in health behaviors and vice versa (30). Given the changing nature of evidence for prevention and potential advances in early detection methods, future research is needed to determine whether these changes affect prevention representations and subsequent behavior.

Healthy behaviors can affect cancer incidence and death rates; therefore, it is important to understand factors influencing these behaviors. Leventhal's Common Sense Model posits that one of these factors is how people perceive cancer controllability. To our knowledge, this study is the first to provide nationally representative data on controllability representations for specific cancers. These results illuminate how lay people think about prevention and detection for 3 common cancers and provide evidence that these representations are related to recommended cancer prevention behaviors. This finding suggests that cancer prevention representations should be addressed in health messages and interventions.

## Figures and Tables

**Table 1:** Demographic and Health Characteristics by Cancer Type, Health Information National Trends Survey, 2005

Demographic and Health Characteristics	Cancer Type,^a^ No. (%)			Total, No. (%)

	Colon	Lung	Skin	
**Sex**				
Male	693 (48.0)	625 (46.0)	611 (50.4)	1,929 (48.1)
Female	1,285 (52.0)	1,247 (54.0)	1,125 (49.6)	3,657 (51.9)
**Mean age, y**	45.6	45.9	44.3	45.3
**Race/ethnicity**				
Hispanic	159 (13.1)	151 (11.5)	186 (14.4)	496 (13.0)
Non-Hispanic white	1,479 (70.9)	1,389 (70.5)	1,235 (68.0)	4,103 (69.9)
Non-Hispanic black	154 (9.5)	146 (9.8)	138 (10.7)	438 (10.0)
Other	97 (6.5)	104 (8.2)	98 (6.9)	299 (7.1)
**Education**				
Less than high school	250 (15.3)	214 (12.7)	223 (15.4)	687 (14.5)
High school graduate	519 (28.3)	476 (30.0)	452 (31.5)	1,447 (29.9)
Some college	405 (25.8)	395 (25.6)	382 (25.0)	1,182 (25.5)
College degree or more	728 (30.5)	721 (31.7)	610 (28.0)	2,059 (30.1)
**Cancer history**				
No cancer history	459 (26.0)	446 (26.6)	374 (24.1)	1,279 (25.6)
Family history	1,170 (62.0)	1,116 (62.2)	1,069 (64.8)	3,355 (63.0)
Personal history	74 (3.0)	69 (3.0)	67 (3.0)	210 (3.0)
**Family and personal history**	246 (9.0)	212 (8.2)	197 (8.0)	655 (8.4)

a Respondents were randomly assigned to answer prevention representation questions about colon, lung, or skin cancer. If respondents had been diagnosed with the specific cancer they were assigned to, they were not asked any prevention representation questions.

**Table 2:** Multivariate Associations^a^ Between Cancer Type and Respondents' Health Beliefs, Health Information National Trends Survey, 2005

Type of Cancer	No. of Respondents	No. of Behaviors Listed, Mean (95% CI)			Respondents' Health Beliefs, % (95% CI)		

		Total Prevention	Primary Prevention^b^	Secondary Prevention^c^	Disagree: Not Much You Can Do to Lower Chances	Agree: Behavior Causes Cancer	Agree: Screening Leads to Early Detection
Colon	1,978	1.34 (1.26-1.42)	0.95 (0.89-1.01)	0.39 (0.35-0.43)	79 (75-83)	48 (44-52)	90 (88-92)
Lung	1,872	1.71 (1.65-1.77)	1.67 (1.61-1.73)	0.04 (0.02-0.06)	82 (80-84)	83 (81-85)	87 (85-89)
Skin	1,736	2.11 (2.05-2.17)	2.03 (1.97-2.09)	0.07 (0.05-0.09)	85 (83-87)	71 (67-75)	88 (86-90)

Abbreviation: CI, confidence interval.

a Multivariate analyses controlled for age, sex, race/ethnicity, education, and cancer history.

b Primary prevention behaviors were the following: For colon cancer, "don't drink alcohol," "don't smoke," "eat fiber," "eat fruits and vegetables," "eat healthy/better nutrition," and "exercise." For lung cancer, "avoid asbestos," "avoid polluted air," "don't smoke/quit smoking," "eat healthy," "exercise," and "stay away from secondhand smoke." For skin cancer, "do not use tanning beds/tanning salons," "stay out of the sun," "wear protective hat/clothing," and "wear sunscreen."

c Secondary prevention behaviors for all 3 cancers were "get screened for cancer/get tested" and "have regular checkups."

**Table 3:** Multivariate Associations Between Prevention Behavior andType of Prevention Representation, Health Information National Trends Survey, 2005^a^

Prevention Behavior	No. of Participants	No. of Behaviors Listed					

		Total Prevention, Mean (95% CI)	*P* Value^b^	Primary Prevention, Mean (95% CI)^c^	*P* Value^b^	Secondary Prevention, Mean (95% CI)	*P* Value^b^
**Has had colonoscopy/sigmoidoscopy^d^**							
Never	411	1.21 (1.09-1.33)	<.001	0.85 (0.73-0.97)	.003	0.37 (0.31-0.43)	.04
>5 years ago	99	1.39 (1.17-1.61)		1.00 (0.82-1.18)		0.39 (0.27-0.51)	
≤5 years	537	1.64 (1.44-1.84)		1.14 (1.00-1.28)		0.50 (0.40-0.60)	
**Fruit and vegetable intake**							
<1 serving/day	131	1.28 (1.02-1.54)	.66	0.93 (0.67-1.19)	.92	0.35 (0.23-0.47)	.16
1-4 servings/day	1,462	1.37 (1.29-1.45)		0.96 (0.88-1.04)		0.41 (0.37-0.45)	
≥5 servings/day	290	1.30 (1.10-1.50)		0.98 (0.82-1.14)		0.32 (0.24-0.40)	
**Smoking status**							
Current	342	1.58 (1.48-1.68)	.01	1.56 (1.46-1.66)	.01	0.02 (0-0.04)	.41
Former	558	1.84 (1.74-1.94)		1.80 (1.70-1.90)		0.04 (0-0.08)	
Never	952	1.75 (1.67-1.83)		1.70 (1.62-1.78)		0.05 (0.03-0.07)	
**Sunscreen use**							
Never	486	1.93 (1.83-2.03)	.004	1.87 (1.77-1.97)	.01	0.06 (0.04-0.08)	.89
Rarely	282	2.11 (1.99-2.23)		2.05 (1.91-2.19)		0.07 (0.01-0.13)	
Sometimes	355	2.19 (2.05-2.33)		2.11 (1.99-2.23)		0.08 (0.04-0.12)	
Often	258	2.29 (2.05-2.43)		2.21 (2.07-2.35)		0.08 (0.04-0.12)	
Always	281	2.16 (1.98-2.34)		2.08 (1.90-2.26)		0.08 (0.04-0.12)	

Abbreviations: CI, confidence interval.

a These analyses controlled for age, sex, race/ethnicity, education, and cancer history.

b Satterthwaite-adjusted *F* tests and their corresponding *P* values were used to determine statistical significance.

c Primary prevention behaviors were the following: For colon cancer, "don't drink alcohol," "don't smoke," "eat fiber," "eat fruits and vegetables," "eat healthy/better nutrition," and "exercise." For lung cancer, "avoid asbestos," "avoid polluted air," "don't smoke/quit smoking," "eat healthy," "exercise," and "stay away from secondhand smoke." For skin cancer, "do not use tanning beds/tanning salons," "stay out of the sun," "wear protective hat/clothing," and "wear sunscreen."

d Analyses with colonoscopy/sigmoidoscopy screening were restricted to respondents aged 50 years or older.

**Table 4:** Multivariate Associations Between Health Beliefs and Behavior, Health Information National Trends Survey, 2005^a^

Prevention Behavior	No. of Respondents	**Disagree: Not Much You Can Do to Lower Chances, % (**95% CI**)**	* P* Value^b^	Agree: Behavior Causes Cancer, **% (**95% CI**)**	* P* Value^b^	Agree: Screening Leads to Early Detection, **% (**95% CI**)**	* P* Value^b^
**Has had colonoscopy/sigmoidoscopy^c^**							
Never	411	70 (64-76)	.37	47 (39-55)	.57	95 (91-99)	.61
>5 years ago	99	78 (66-90)		49 (37-61)		93 (87-99)	
≤5 years	537	75 (69-81)		52 (46-58)		92 (88-96)	
**Fruit and vegetable intake^c^**							
<1 serving/day	131	74 (64-84)	.42	46 (32-60)	.77	95 (93-97)	.38
1-4 servings/day	1,462	78 (74-92)		48 (44-52)		90 (88-92)	
≥5 servings/day	290	84 (74-94)		51 (39-63)		88 (78-98)	
**Smoking status^c^**							
Current	342	80 (74-86)	.78	77 (71-83)	.03	85 (79-91)	.40
Former	558	83 (77-89)		82 (76-88)		89 (85-93)	
Never	952	83 (79-87)		86 (8290)		87 (85-89)	
**Sunscreen use^c^**							
Never	486	84 (80-88)	.30	71 (65-77)	.60	86 (82-90)	.48
Rarely	282	85 (79-91)		67 (57-77)		92 (8698)	
Sometimes	355	89 (85-93)		68 (60-76)		89 (81-97)	
Often	258	89 (83-95)		75 (65-85)		85 (77-93)	
Always	281	81 (73-89)		76 (68-84)		87 (81-93)	

Abbreviations: CI, confidence interval.

a These analyses controlled for age, sex, race/ethnicity, education, and cancer history.

b Satterthwaite-adjusted *F* tests and their corresponding *P* values were used to determine statistical significance.

c Analyses with colonoscopy/sigmoidoscopy screening were restricted to respondents aged 50 years or older.
